# Supplementary material for: Are carbon emissions trading and green financial instruments synergistic? -Comprehensive quantitative research based on content analysis
Source: PLoS One. 2024 Mar 7;19(3):e0298601. doi: 10.1371/journal.pone.0298601 (PMC10919601; doi:10.1371/journal.pone.0298601)
Supplement: S1 Data — (ZIP) [file pone.0298601.s001.zip › Data/地级市Z_F工作报告.zip!/░▓╗╒/┴∙░▓╩╨/└┤╫╘╣1⁄2╓┌║┼í╛┬φ┐╦╩2╛▌═°í┐_2016.docx]

各位代表：
　　现在，我代表市人民政府，向大会报告工作，请予审议，并请市政协委员和其他列席人员提出意见。
　　一、  团结奋斗，“十二五”发展迈上新台阶
　　“十二五”是六安绿色发展、奋力崛起的重要时期。五年来，全市人民在市委的坚强领导下，认真贯彻落实党的十八大、十八届三中、四中、五中全会和习近平总书记系列重要讲话精神，积极应对各种风险和挑战，坚持稳中求进、改革创新，全力提质增效、转型升级，经济社会发展取得长足进步。
　　这五年，综合实力显著增强。生产总值迈上千亿元台阶，达到1143亿元，年均增长9.2%。财政收入迈上百亿元台阶，达到150亿元，年均增长16.9%。规模工业企业由598户增至1079户。规模工业增加值由246.7亿元增至429亿元，年均增长13.5%，工业化率由34.5%提高至38.5%；社会消费品零售总额由284.7亿元增至562.6亿元，年均增长14.2%；固定资产投资由517.9亿元增至1131亿元，年均增长21%。贷款余额由484亿元增至1082亿元，年均增长17.5%。三次产业结构由23.5:42.3:34.2调整为19.5:46.1:34.4。粮食生产连年丰收，荣获全国粮食生产先进市和全省粮食生产“三大行动”先进市。
　　这五年，绿色发展扎实推进。积极实施绿色发展战略，着力推进“一心一廊”、“一谷一带”平台建设，被列为国家级生态示范区、生态环境监察试点市和环保部绿色GDP2.0试点市。完成节能减排主要任务，节约集约用地水平不断提升，金寨县积极创建国家级国土资源节约集约模范县。实施森林增长工程113万亩，基本完成百万亩油茶产业发展行动计划。旅游业加快发展，天堂寨成功创建5A级旅游景区，全市拥有4A级以上景区25家、省级最佳旅游乡镇19个，数量分别居全省第二和第一。现代物流、金融保险、电子商务等现代服务业加快发展，“互联网+”快速兴起，“特色中国•六安馆”上线运营，霍山、舒城、金寨县入选国家电子商务进农村综合示范县。科技创新能力不断增强，省级以上创新型企业、知识产权优势企业、工程技术研究中心分别达24家、7家、19个，发明专利累计授权量516件，荣获全国科技进步先进市。
　　这五年，城乡面貌深刻变化。城乡规划体系不断完善，在全省率先完成各县总规修编。中心城区累计实施重点工程190项，完成政府性投资180亿元，建成区面积扩展到74.4平方公里、人口增加到66.2万人，荣获国家园林城市、省级文明城市等；县城“三治三增三提升”行动深入开展，集镇建设加快，城镇空间不断拓展，配套设施逐步完善，预计城镇化率由35.9%提高至42.7%。累计投入24.9亿元，推进248个省级美好乡村中心村建设，“三线三边”城乡环境整治成效显著。现代综合交通体系加快建设，阜六铁路、宁西铁路复线和沪陕、六武、周六、六潜高速等建成通车，铁路、高速公路通车里程分别达336公里、370公里；新建改建国省干线公路1005公里、农村公路3500公里、改造危桥350座，大别山旅游快速通道基本建成，公路总里程居全省首位，累计投入230亿元，是“十一五”时期的6.6倍。水资源保障体系不断完善，治淮3项骨干工程全面完成，淠河、史河治理工程加快实施，治理水土流失面积736平方公里。加强耕地保护，累计完成高标准基本农田建设302万亩。防汛抗旱、供电、通讯等保障能力不断增强。
　　这五年，改革开放不断深化。行政区划调整顺利推进，叶集行政区获国务院批复。文化体制改革成效显著，连续三年荣获全国文化体制改革先进市。农村金融、科技、农村土地等改革实现突破，财税、医药卫生、投融资和商事制度改革不断深化。对外开放持续扩大，累计招商到位内资3268亿元、外资15.6亿美元，年均增长22.3%、22.7%；累计实现进出口总额35亿美元，年均增长6.4%。合作交流力度加大，成功举办三届中国（六安）国际羽绒博览会、四届大别山（六安）山水文化旅游节、绿色发展跨洲视频连线研讨会、中蒙俄万里茶道（国际）协作体2015会议。积极融入合肥经济圈，全面实施皖江示范区建设，15个省级开发区（含筹建）建成区面积158平方公里，六安开发区升级为国家级经济技术开发区，霍邱、叶集经济开发区获批全国循环化改造示范试点园区。检验检疫、海关办事机构组建运营。
　　这五年，社会事业全面发展。公共文化服务体系进一步完善，累计建成农民文化乐园27个、乡镇综合文化站157个、农家书屋2563个，公共文化场馆免费开放，霍山县荣获全国文化先进县。文明创建活动扎实开展，公民文明素质和社会文明程度稳步提高，累计入列“中国好人榜”29人。提前完成学前教育第一个三年行动计划并启动第二个三年计划，改造农村薄弱学校679所，完成义务教育标准化学校建设1555所，中心城区基础教育学校设施大为改善，霍山县、金安区通过义务教育均衡县（区）国家验收。职业教育加快发展，六安职业技术学院通过省首批地方技能型高水平大学立项评审，大别山职教集团成立，金寨职业学校建成使用。公共卫生、医疗服务体系进一步完善，看病难看病贵得到缓解。人口低生育水平保持稳定，荣获全省计划生育先进市。全民健身运动广泛开展，成功举办第十五届亚洲赛艇锦标赛等重大赛事。“六五”普法顺利完成，平安六安建设扎实推进，先后荣获全省综治（平安建设）先进市和进步市，霍山县再获“长安杯”。国防动员、后备力量建设稳步发展，再获全国双拥模范城。广播电视、新闻出版、统计、审计、信访、档案、地方志、民族宗教、老龄、妇女儿童、社会慈善等事业得到加强，气象、地震、防灾减灾、人民防空等工作取得新进展。
　　这五年，人民生活持续改善。预计城镇、农村居民人均可支配收入分别达到22260元、9116元，年均增长11.5%、14.2%。累计投入民生领域资金1188亿元，民生工程实施工作连续多年全省领先。城镇累计新增就业26.6万人，登记失业率控制在4.5%以内。社会保障体系更加健全，城镇职工五大保险参保率逐年递增，城乡居民社会养老保险、医疗保险实现全覆盖，城乡低保应保尽保。建设各类保障性安居工程14万套，完成农村危房改造10.5万户。以实施“5+1”项目为契机，抓金寨、带全市，加大扶贫开发力度，累计减少贫困人口73.7万人，贫困发生率由20%降至8.6%。
　　这五年，政府建设切实加强。政府职能加快转变，市县乡三级政府权力清单、责任清单和涉企收费清单制度全面建立，市级行政审批事项减至188项，保留市级收费94项。完成新一轮市县政府机构改革。健全政府决策机制，把公众参与、专家论证、风险评估、合法性审查、集体讨论决定作为重大行政决策法定程序。自觉接受人大依法监督、政协民主监督和社会舆论监督，共办理人大议案10件、代表建议763件和政协委员提案1065件。加强政府权力运行监管，深化政务公开，强化行政监察，推进审计监督全覆盖，连续四年在全省依法行政考评中位居前列。加强机关效能建设，健全工作落实机制。深入开展党的群众路线教育实践活动，全面践行“三严三实”，加大党风廉政建设和反腐败力度，政府执行力和公信力进一步提升。
　　各位代表！
　　刚刚过去的2015年，是“十二五”收官之年。一年来，面对复杂宏观环境和经济下行压力，我们主动适应经济发展新常态，坚持绿色发展战略，统筹做好稳增长、促改革、调结构、惠民生、防风险各项工作，保持了经济社会稳定健康发展。全市生产总值增长7%，财政收入增长5.6%，社会消费品零售总额增长10.9%，预计城镇、农村居民人均可支配收入分别增长8%、10%。主要做了以下工作：一是抓工业稳增长。全面贯彻落实中央及省稳增长各项政策措施，支持实体经济和小微企业发展，规模工业增加值增长4.8%。扎实开展“技术改造年”活动，完成工业投资455.2亿元，增长5.6%；其中技改投资243.5亿元，增长46.5%。强化精准帮扶，兑现工业发展专项资金1.2亿元，减免缓抵各项税费22.6亿元。加快两化融合步伐，培育国家级两化融合管理体系贯标试点企业1家。中心城区工业企业“退城进园”工作稳步推进。二是抓投入添后劲。健全完善领导精准帮扶重点企业和重点项目推进机制，全面推行“四督四保”制度，推动项目工作提质提效。完成固定资产投资1131亿元，增长12.7%；其中，亿元以上重点项目完成投资678.8亿元，增长11.3%。强化产业招商、合作招商和以商招商，到位内资455亿元，增长11%；利用外资3.87亿美元，增长10%。金寨抽水蓄能电站、万达城市综合体、亚夏汽车城、胜利精密电子、联胜产业公园、吉宝冷链物流等项目加快实施；中财管道一期、江淮高效节能电机、金寨垃圾发电等项目竣工投产。畅通金融渠道，扎实推进“4321”新型政银担合作，开展“税融通”业务，全年新增贷款123.8亿元；实现直接融资36.5亿元，迎驾贡酒在上交所成功上市，亿民照明在“新三板”挂牌，24家企业在区域性股权交易市场挂牌。中信银行、招商银行入驻六安。三是抓转型调结构。围绕调结构转方式促升级，谋划推进十大重点工程。大力实施创新驱动，新认定国家高新技术企业30家，新增省级创新型企业8家；申请发明专利1720件，增长56%；授权发明专利267件，增长147%。实现高新技术产业增加值89.5亿元，增长35.3%；战略性新兴产业产值248.5亿元，增长6.4%。实施旅游业重点项目三年行动计划，全年共接待游客2926万人次，实现旅游综合收入202亿元；万佛湖景区5A创建通过专家评审，霍山县荣获国家级休闲农业和乡村旅游示范县。四是抓农业夯基础。开展粮食绿色增产模式攻关，推进高标准农田建设、农业综合开发和小型水利工程改造提升，连续17年实现基本农田占补平衡有余，粮食总产491.3万吨。加快推进农产品加工“168”工程，实现加工产值860亿元。积极构建新型农业经营体系，新增亿元以上龙头企业8家、农民专业合作社1183家、家庭农场1642户。大力发展品牌农业，新认证国家地理标志保护产品1件、国家地理标志证明商标6件；全市农业“三品”认证54件，总量居全省第一。全面落实强农惠农政策，发放各项补贴资金35亿元。五是抓改革增动力。继续深化行政审批制度改革，政府权力清单运行平台加快建设。探索实施政府购买服务，谋划政府与社会资本合作（PPP）项目115个。完成全市不动产统一登记机构职责整合。理顺市直行政事业单位国有资产管理体制，事业单位改革加快推进，市直国有企业分类改革主体工作基本完成。机关事业单位工资制度改革和公车制度改革顺利实施。深化商事制度改革，全面实施“三证合一、一照一码”，新登记各类市场主体2.8万户。积极开展农村集体产权制度改革试点，扎实推进农村土地承包经营权确权登记颁证工作，启动金寨县国家级农村宅基地改革试点。有序流转耕地295万亩，占全市耕地面积45.2%。金安、裕安区成功开展全国农业水价综合改革试点。全面推进农村金融改革，7家农商行改制成功，7家村镇银行开业运行。继续深化医药卫生体制改革，全面启动城市公立医院综合改革。六是抓城乡促协调。积极推进新型城镇化，成功创建国家可再生能源建筑应用示范城市、省节水型城市、省建筑产业现代化综合试点城市，探索开展海绵城市、智慧城市建设。加快中心城区建设，共实施重点项目87个、完成政府性投资39亿元。大力推进征迁工作，中心城区市重点工程完成征迁202万平方米。加快推进各县县城和叶集城区建设，全面拓展框架，畅通对外交通，承载能力大幅提升，人居环境明显改善。分类推进小城镇建设，舒城县实施以“六个一”工程为核心的集镇建设，金寨县天堂寨镇入选全国特色景观旅游名镇示范名录，裕安区独山镇、金安区张店镇列入全省美丽宜居小镇名录。大力推进美好乡村建设，第二批87个省级中心村建设全部完成。加强合肥经济圈和皖北结对合作，推进社保一体化、公积金异地互认和转移接续等5项合作专题，实施霍邱、舒城县与合肥市15项结对合作事项。规范土地管理，顺利通过国家土地例行督察。七是抓民生构和谐。大力实施37项民生工程，投入资金133亿元，增长21%。坚持精准扶贫，完成重点贫困村、贫困人口建档立卡工作，共投入扶贫专项资金4.9亿元，实施扶贫项目1700多个，预计全年减少贫困人口14万。深入开展大气污染防治，空气质量持续改善，城区烟花爆竹限放工作得到巩固。扎实开展大别山区（淠河流域）水环境生态补偿工作，全市集中式饮用水源地水质达标率100%。完善安全生产责任体系，着力开展重点行业、领域隐患排查和专项整治，安全生产形势总体平稳。加强社会治安综合防控体系建设，改进信访维稳工作，启动社会服务管理信息化工程，加快建设城区报警与视频监控系统、消防基础设施，成功处置“3.11”劫持人质案件，人民群众安全感不断提升。
　　各位代表！
　　回顾五年发展，我们倍感欣慰。“十二五”时期是六安发展历程中最为困难的时期之一。五年来，面对着振兴发展、脱贫攻坚、环境保护的异常艰巨历史任务，面临着传统动力逐渐消退、新生动能尚未形成、下行压力持续加大的异常复杂宏观环境，我们保持战略定力，主动适应新常态，创造性地提出了绿色发展理念，砥砺奋进，攻坚克难，全力以赴扩总量、调结构、提质量，不遗余力打基础、补短板、促平衡，为六安奋力崛起奠定了良好基础。这是省委、省政府和市委正确领导的结果，是市人大、市政协及社会各方面大力支持的结果，是全市广大干部群众团结奋斗的结果。在此，我代表市人民政府向大家表示诚挚的感谢!
　　在肯定成绩的同时，我们也要清醒地看到存在的困难和问题。主要是：发展速度不快、结构不优、质量不高问题仍较突出；有效需求不足，供给效率不高，传统产业困难加大，战略性新兴产业支撑不足，现代服务业发展相对滞后，农业产业化水平不高；经济下行压力加大，去年生产总值、财政收入、规模工业增加值等指标增幅没有达到预期目标；基本公共服务供给不足，文化、教育、卫生、社会保障等方面存在一些短板，全市仍有46万贫困人口，脱贫攻坚任务十分艰巨；环境保护、安全生产、食品药品安全等方面还存在不少隐患；政府工作执行力、管理服务效能仍存在薄弱环节，“四风”问题还不同程度存在，违纪违法现象还时有发生。对此，我们要高度重视，认真研究解决。
　　二、奋力拼搏，全面建成小康六安
　　“十三五”是全面建成小康社会的决战阶段，是调结构转方式促升级的关键时期。我们既面临着发展大势总体向好、政策机遇多重叠加、发展基础日渐积累、发展条件明显改善等有利因素，也存在着宏观环境依然复杂、下行压力仍在持续、新旧动能转换艰难等不利条件，发展的任务艰巨而繁重。站在新的历史起点，我们一定要适应新常态、把握新常态、引领新常态，积极抢抓机遇，有效应对挑战，保持战略定力，坚定信心决心，推动经济社会更好更快发展。
　　根据市委三届十一次全会通过的《关于制定国民经济和社会发展第十三个五年规划的建议》，我市“十三五”发展的指导思想是：高举中国特色社会主义伟大旗帜，全面贯彻落实党的十八大和十八届三中、四中、五中全会精神，以马列主义、毛泽东思想、邓小平理论、“三个代表”重要思想、科学发展观为指导，深入贯彻习近平总书记系列重要讲话精神，认真落实“五位一体”总体布局和“四个全面”战略布局，坚持发展第一要务，坚持以绿色发展为总取向、以稳中求进为总基调、以调转促为主抓手、以改革创新为主动力，大力实施开放合作战略、新型工业化战略和新型城镇化战略，加快“一心一廊”、“一谷一带”建设，着力提升经济总量，优化经济结构，合力脱贫攻坚，保障改善民生，走出一条大别山革命老区绿色振兴的特色之路，确保如期全面脱贫，确保如期全面建成小康社会。
　　在市委《建议》指导下，市政府编制了《“十三五”规划纲要》（草案）。综合考虑，提出今后五年全市经济社会发展的主要目标（以下数据均不含寿县）：
　　——综合实力再上台阶。生产总值年均增长8.5%左右，经济总量超过1500亿元，基本建成全国重要的农产品生产加工基地、全国知名的红色旅游胜地、全国重要的文化生态休闲旅游目的地和大别山革命老区核心增长极。
　　——产业结构明显优化。三次产业结构调整到15:48.5:36.5，战略性新兴产业产值占工业总产值比重提高到18%，现代服务业增加值占服务业比重提高到40%，农产品加工产值与农业产值之比达到2.5，产业集群集聚作用更加凸显，科技进步对产业增长的贡献率明显提升，产业转型升级步伐加快。
　　——质量效益显著提高。财政收入超过205亿元。主营业务收入超百亿元企业2-3家，超10亿元30-40家。新增中国驰名商标15件以上，总数达28件以上；新增安徽省著名商标150件以上，总数达280件以上；新增安徽省名牌产品40个以上，总数达140个以上。
　　——城乡发展统筹协调。中心城市首位度和承载能力显著提升，叶集区实现加快发展，县城和重点城镇带动力明显增强，一廊、一谷、一带建设加快推进，美丽乡村建设全面完成，城乡基础设施支撑能力显著增强，城镇化率达到48.4%。
　　——生态环境优势凸显。绿色发展统领作用充分发挥，主要环境指标达到全省领先水平，森林覆盖率达到47%，基本建成安徽优质水源地、大别山区转型发展示范区、全国绿色发展和生态文明建设先行区，全面构筑起长江和淮河中下游地区重要的生态安全屏障。
　　——贫困人口全面脱贫。实施产业扶贫、就业扶贫、健康扶贫、社保兜底扶贫等工程，稳定实现贫困人口“两不愁、三保障”，到2018年实现442个贫困村全部出列，2019年6个贫困县区全部摘帽、46万贫困人口全部脱贫。
　　——小康社会全面建成。城乡居民就业创业空间更加宽广，增收渠道更加多样，收入与经济同步增长，城镇和农村居民人均可支配收入分别达到35000元和15000元。社会事业全面发展，基本公共服务均衡化均等化水平不断提高。社会保障能力持续增强，民主法制建设取得积极进展，社会管理水平明显提升，社会更加包容和谐，幸福六安建设迈出坚定步伐。
　　实现上述目标，今后五年工作的主要任务是：
　　一是加快绿色发展。坚持绿色发展总战略，将绿色理念贯穿到发展各领域和全过程。科学谋划空间开发格局，加快推进“一心一廊”、“一谷一带”等绿色发展平台建设。统筹全市发展现状与未来发展战略布局，做好顶层设计，强化规划引领。充分发挥生态资源优势，着力打造绿色生态产业体系。继续加强水土流失治理，推进新一轮退耕还林还湿工程和绿色长廊建设，加强自然保护区、风景名胜区、生态功能区和矿产资源开发区的生态保护、建设与修复。实行最严格水资源管理制度，完善大别山区（淠河流域）水环境生态补偿机制。大力推进大气污染、水污染、土壤污染等防治工作，加大节能减排力度，严守生态底线，严格环保执法。
　　二是着力转型升级。全力实施中国制造2025，推进互联网+，加快工业化与信息化深度融合，引导企业加快智能化和绿色化改造步伐。促进战略性新兴产业集聚发展，力争到2020年，打造10个左右市级战略性新兴产业集聚发展基地，基地工业总产值突破千亿元。提升现代服务业，建设旅游经济强市，积极培育健康养生产业，促进文化体育旅游融合发展。大力发展电子商务，打造陆路型物流节点城市。全面推进优质粮油、畜禽、水产、茶叶、水果等主导农产品基地建设，积极构建新型农业经营体系，大力发展特色农产品精深加工，提高农业质量和效益。
　　三是实施创新驱动。全面深化改革，农业农村、医药卫生体制、财税金融、国有资产管理等重点领域改革取得明显成效，市场在资源配置中的决定性作用更加突出，政府作用更好发挥，发展活力不断激发。推动体制创新、科技创新、管理创新，争创省级创新型试点城市。引导和支持企业加大研发投入，加快企业主体、创新载体和产学研一体建设，打造一批引领产业高端发展的创新型龙头企业和高新技术企业。深入推进大众创业、万众创新，拓展众创空间，推广创客空间等新型孵化模式。进一步放开投资领域，推出更多面向社会资本的投资项目。
　　四是统筹协调发展。推进以人为核心的新型城镇化，深化户籍制度改革。统筹推进中心城市、县城建设，完善城市功能，提升品质特色，充分发挥其辐射带动作用，全面推进小城镇和美丽乡村建设。加大基础设施投入，完善综合交通运输、能源、水利保障和信息网络体系，促进城乡公共资源均衡配置。加强历史文化保护，重视非物质文化遗产传承，建设红色文化胜地，做大做强文化产业，打响六安文化品牌。积极打造红色土地上新的精神高地，探索红色土地、绿色发展和精神文明建设协调发展的新路径。
　　五是扩大开放合作。抢抓“一带一路”、长江经济带、大别山革命老区振兴发展等重大战略机遇，积极参与长三角一体化，全面融入合肥经济圈，加快承接产业转移，强化大别山区域合作。大力实施精准招商，突出产业链招商、以商招商。建立产业引导基金，通过股权投资等市场化方式，大力引进社会资本，加快提升产业规模和发展水平。加强检验检疫、海关机构建设，设立海关特殊监管区域，提高贸易便利化水平。建设加工贸易出口和绿色农产品出口基地，创建出口品牌，培育外贸竞争新优势。支持企业引进国外先进设备、技术。鼓励有条件的企业“走出去”。
　　六是实现成果共享。巩固提升民生工程，着力保障改善民生。坚持优先发展教育，完善学前教育公共服务体系，均衡配置义务教育资源，普及高中阶段教育，实施现代职业教育体系建设规划。完善创业就业扶持政策，加强就业服务体系建设，推行终身职业技能培训制度。健全工资水平正常增长机制、支付保障机制，完善最低工资增长机制，推行企业工资集体协商制度。实施全民参保计划，进一步完善城乡居民基本养老保险制度，全面建立统一的城乡居民基本医疗保险制度。统筹社会救助体系、医疗卫生服务体系和养老服务体系建设，促进人口均衡发展，提高全民健康水平。深化网格化管理，加强社会服务管理信息化建设。
　　各位代表！
　　六安作为集老区、山区、库区、行蓄洪区为一体的欠发达地区，脱贫攻坚任务十分艰巨，责任重于泰山。我们必须把脱贫攻坚作为“十三五”期间头等大事和第一民生工程，坚持以脱贫攻坚统揽经济社会发展全局，坚持精准扶贫、精准脱贫，以更坚定的决心、更明确的思路、更精准的举措、更严实的作风，举全市之力，聚各方之智，坚决打赢脱贫攻坚战，确保2020年如期全面建成小康社会。
　　三、开拓创新，扎实抓好2016年各项工作
　　今年是“十三五”规划的开局之年，也是推进结构性改革的攻坚之年。我们要适应经济发展新常态，坚持稳中求进工作总基调，坚持稳增长、调结构、惠民生、防风险，坚持宏观政策要稳、产业政策要准、微观政策要活、改革政策要实、社会政策要托底，以调转促为主抓手，着力加强结构性改革，在适度扩大有效需求的同时，着力去产能、去库存、去杠杆、降成本、补短板，增强持续增长动力，保持经济社会稳定健康发展。主要预期目标是：全市生产总值增长7.5%左右，财政收入增长7%左右，规模工业增加值增长7.5%左右，固定资产投资增长12%左右，社会消费品零售总额增长11%左右，城镇、农村居民人均可支配收入分别增长7.5%和9.5%左右，减少贫困人口13万左右，城镇登记失业率控制在4.5%以内，人口自然增长率控制在9‰以内，出生人口性别比保持平稳下降趋势，完成节能减排目标任务。
　　为此，着力抓好八个方面工作：
　　（一）推进供给侧结构性改革，提高供给体系质量和效率。
　　提升改造传统产业。着力培育装备制造、绿色食品加工、新能源、生物医药、电子信息、采矿冶金及新材料等六大主导产业，力争产值占规模工业比重超过62%。支持传统产业实施智能化改造，引导企业开拓绿色产品、推广节能和资源综合利用。落实企业税费减免政策措施，开展降本增效专项行动，对资不抵债、扭亏无望的“僵尸企业”实行兼并重组、破产清算，提升企业效益和资源配置效率。继续实施精准联系帮扶重点企业制度，进一步解决中小企业融资、用地、用工等难题。
　　加快战略性新兴产业集聚发展。以培育壮大龙头企业、增强创新能力为抓手，推进产业链招商，完善产业配套。集中力量建设霍山高端装备关键零部件产业、舒城电子信息配套产业、金寨清洁能源产业、六安新能源（汽车）制造及零配件生产、裕安循环经济及新材料生产、霍邱铁基新材料研发生产集聚基地，着力打造叶集中国中部家居产业园，谋划建设金寨钼基新材料研发生产集聚基地。
　　大力发展现代服务业。推动现代物流、金融保险、工业设计、科技服务等生产性服务业向专业化、高端化发展。积极培育云计算、大数据、物联网等新兴业态，大力发展健康养生、文化教育、体育健身等产业，推进生活性服务业品质化、精细化。继续实施旅游业发展三年行动计划，加快五大旅游区、四条特色旅游线路、旅游集散中心体系和旅游信息平台建设。大力发展住房租赁市场，全面推行房屋征迁安置货币化；改善供给结构，完善功能配套，激活存量，管控增量，促进房地产业平稳健康发展。
　　优化农业供给结构。调整优化产业结构、种养结构和品种结构，提高经济作物比重。深入开展粮食绿色增产模式攻关，推进300万亩优质稻米生产核心区建设。依据资源优势、生产习惯和市场需求，提倡地力较差的耕地实行休耕轮作制度，推广适宜发展的经济林果、设施蔬菜、苗木花卉和中药材。推进“菜篮子”生产基地建设，增加蔬菜、瓜果、禽蛋有效供给。推广畜牧业低碳循环养殖和渔业健康养殖技术，全面提升规模化、集约化、标准化养殖水平。
　　（二）加快调转促步伐，强力推进绿色发展。
　　强化创新驱动。引导和支持企业加大科研投入，培育省市级企业技术中心15家、省级工业设计中心2家。加强政策引导，提高专利创造质量，力争全年发明专利申请量1500件以上、授权量150件以上。加强科技人才和企业家队伍建设，完善科技成果转化激励机制。创新人才流动机制，重点引进高层次人才和创新团队。加快六安大学科技园建设，拓展众创空间，为创业创新提供低成本、便利化的综合服务。
　　加快绿色平台建设。坚持高水平规划引领，大力推进“一心一廊”、“一谷一带”建设。科学布局中心城市空间，完善城市绿道、步道设施，推广公共交通和绿色出行，开展海绵城市和综合管廊建设试点。推动合六叶工业走廊交通、能源、公共服务等一体化建设，围绕装备制造、汽车零部件、机电家电、新能源、木竹加工，打造一批百亿级产业集群。充分发挥六安茶谷纽带作用，加快沿线乡镇一体化建设，大力发展生态农业、绿色食品加工和休闲观光农业。加快淠河生态经济带基础设施建设，扎实开展全流域治理，推进大别山湿地公园建设，着力发展优质高效农业和旅游文化业。
　　加强生态环境建设。深入开展大气污染防治专项行动，完善减排统计、监测和考核三大体系建设。强化水污染、土壤污染防治，加快推进各县区、开发区污水处理厂建设。加强重点流域水环境综合治理，实行最严格水资源管理制度。继续推进森林增长工程和绿色长廊建设，确保国家森林城市创建工作通过验收。落实能源消费强度和总量目标责任，严格实施项目能评制度。加快节能技改重点项目建设，推广节能产品，推动重点行业节能降耗。开展农村生活垃圾治理三年行动，逐步改善农村人居环境。推动绿色建筑规模化发展。
　　加快园区转型升级。坚持集群发展、绿色集约、产城融合、示范带动，优化开发园区空间布局，明晰首位产业和主导产业，着力完善基础设施和配套服务业。支持六安开发区创建国家产城融合示范区和转型升级示范区，鼓励市示范园区申报国家级高新技术产业基地，加快霍邱、叶集经济开发区循环化改造试点项目建设。创新园区开发模式，引入社会资本开发经营工业地产、建设园中园。开展闲置和低效用地专项清理行动，提高土地利用效率、投资强度和产出率。加强信息化基础设施建设，全面推进15个“智慧园区”创建工程。加快推进中心城区工业企业“退城进园”步伐。
　　（三）扩大有效需求，增强经济发展动力。
　　提升投资效益。以有效投入促进有效供给，全年新开工亿元以上项目130个以上、建成70个以上。力促霍邱300万吨钢铁深加工项目复工建设，加快应流航空产业园、吉宝冷链物流园、金寨新能源制造、中鑫模具产业园、合六南通道、淠河治理等项目建设，积极推进青年汽车产业园、迎驾野岭产业园、中植新能源汽车生产基地、星瑞汽车变速器、六安传化公路港、德上高速舒城段、六安火车站站房、合安九铁路舒城段等项目尽快开工。大力推进招商引资，围绕首位产业和主导产业，紧盯重点区域、园区，深化与央企、省企、知名民企合作交流，实现到位内资和利用外资分别增长10%以上。着力加强保障服务，全面实施重大项目建设“四督四保”制度，切实抓好项目落地，推动项目建设。
　　促进消费升级。开展改善消费品供给专项行动，扩大大众旅游消费、养老健康消费和信息消费，增加中高端医疗、文化、体育等服务供给，促进智能家居、数字媒体等新型消费品发展。继续组织开展“文化惠民消费季”和“书香六安•悦读人生”全民阅读活动。深入推进“五位一体”便民服务体系建设，培育壮大商贸龙头企业，大力发展限上企业。认真实施中心城市大型商品交易市场发展专项规划，整合、搬迁、新建、提升一批商品交易市场。大力发展电子商务、物流快递、连锁经营等现代流通方式，积极支持淘宝“特色中国•六安馆”、“蜂狂购”、“邮乐农品•大别山馆”、新华大宗安徽运营中心等电商平台发展，推动六安快递（电商）物流园等标准化、规范化建设。
　　稳定外贸发展。充分利用国际市场开拓、出口信用保险等政策资金，推动企业扩大出口。加强外贸基地建设，促进传统优势外贸产业做大做强，努力扩大电子、汽车零部件、新兴照明等产品出口。积极发展进口贸易，支持企业扩大先进技术设备、关键零部件进口。争取检验检疫机构尽快建设运营、海关设立正式机构，加快保税物流中心、公共保税仓库申报建设进度。
　　拓展融资渠道。加大“税融通”推广力度，发挥续贷“过桥”资金作用。建立市县融资担保风险补偿专项资金，鼓励和引导银行、担保机构扩大“4321”模式应用范围，力争全年新增贷款130亿元。积极对接多层次资本市场，争取新增上市企业1家、“新三板”挂牌企业5家、区域性股权交易市场挂牌企业20家，大力支持企业通过债券市场融资，力争直接融资30亿元以上。大力推进政府和社会资本合作（PPP）模式，引导和撬动社会资本参与基础设施等政府投资项目。
　　（四）继续深化改革，激发发展活力。
　　大力推进行政体制改革。持续推进“放管服”结合，加快建设标准统一、资源共享、业务协同的清单运行平台，推广“互联网+政务服务”。完善公共资源交易电子化平台建设，推进公共资源交易全流程、全范围电子化，实现网上投标、远程评标。深化商事制度改革，进一步放宽市场准入管制。加快事业单位分类改革，全面完成市直生产经营服务类事业单位转企改制。继续深化市场监管体制改革，在交通运输、城乡建设等领域探索推行综合执法。
　　继续深化农村综合改革。全面完成农村土地承包经营权、农村集体土地所有权、集体建设用地使用权确权登记发证工作。开展土地经营权抵押、担保等试点，稳妥推进土地承包经营权、农民住房财产权抵押贷款试点。引导推动农村土地流转和适度规模经营，建立完善流转登记制度，探索工商资本租赁农地的监管和风险防范机制。深入推进金寨农村宅基地制度改革试点，探索宅基地有偿退出机制。加快农村集体产权制度改革进程，建设农村产权流转交易市场。全面实施农业补贴“三合一”改革，开展省级农村集体资产股份合作制改革试点。全面启动农业生产社会化服务示范创建活动。深化林业和水利改革，完善集体林权制度，健全小型水利工程长效管护机制。
　　加快财税金融改革。完善政府预算体系，实行中期财政规划管理，建立跨年度预算平衡机制；加强综合治税信息平台建设，完善协税护税机制；稳妥实施国税、地税征管体制改革；推进营改增改革。创新财政支持发展方式，向市工投公司增加注资7亿元，成立产业发展引导基金；向市融资担保公司增加注资3亿元，支持其做大做强。深化市直行政事业单位国有资产改革，规范资源性国有资产运营管理。深化农村金融综合改革，鼓励农村商业银行增资扩股和上市挂牌，支持金寨县打造改革“升级版”，推动金安区拓展改革领域，促进其他县区改革取得重要突破。
　　推进社会事业改革。深化文化体制改革，鼓励社会力量和社会资本参与公共文化服务体系建设。深化教育领域改革，大力发展普惠性学前教育；推进区域内城乡实施统一的教师配置、办学条件，均衡配置城乡义务教育资源。推进医药卫生体制改革，深化公立医院综合改革，全面实行公立医院药品集中带量采购；建立健全重特大疾病保障制度，深化医保支付制度改革；稳步推进分级诊疗制度建设。实施机关事业单位养老保险制度改革，整合城乡居民基本医保和新农合制度。
　　（五）大力发展现代农业，推动农业可持续发展。
　　发展现代生态农业。加强耕地保护，推进农田水利、土地整治、中低产田改造和高标准农田建设。加快实施农业现代化推进工程，深入开展省级现代生态农业产业化示范市、示范县建设。发展特色农业，集中力量打造优质粮油、蔬菜、畜禽、茶叶、竹木、中药材等6个百亿元产业集群。加强农业资源节约和综合利用，大力推广节地、节肥、节药、节水生产技术和模式，加快发展循环农业，提高农业资源利用效率和产出率。
　　构建新型农业经营体系。加快培育现代农业产业化联合体，构建以龙头企业为核心、农民合作社为纽带、专业大户和家庭农场为基础的新型农业经营组织联盟。引导发展土地股份、农村社区、资金互助、联合社等新型合作组织，支持专业大户发展为家庭农场，培育新型职业农民。新增农民专业合作社400家、家庭农场250家，新培育省级农业产业化示范联合体10家。
　　推进农产品质量体系建设。加强农产品质量安全监管，支持金寨、霍山县争创国家级和省级农产品质量安全县。加强农产品质量安全诚信体系、标准体系和监测评估体系建设，开展食用农产品质量安全可追溯试点。加快发展品牌农业，开展国家级、省级蔬菜、茶叶、水果等标准园和畜禽、水产标准化养殖示范场创建，新增部省级“三园两场”数量10个，农产品“三品”认证数量20个。
　　（六）加快新型城镇化建设，统筹城乡一体化发展。
　　优化城镇体系。按照建设大别山区域中心城市、合肥经济圈副中心城市、长三角城市群节点城市定位，进一步优化城镇发展空间布局和形态。结合行政区划调整，启动城市总规修编，完善控制性详细规划和各类专业规划，强化城市设计。积极开展“多规合一”、海绵城市、智慧城市等专项试点。深化户籍制度改革，有序推进农业转移人口市民化。推进与合肥市交通基础设施、市场一体化等合作专题，加快六安-合肥城际轨道交通前期工作。
　　做大做强中心城市。巩固深化省级文明城市创建成果，深入开展“五城同创”活动。继续实施中心城区重点工程，计划完成政府性投资37亿元，重点推进赤壁路桥等69个项目建设。加快老城区的城中村、棚户区、老旧小区改造，规划建设停车场、菜市场等配套设施，规范物业管理与服务，切实改善人居环境。有序推进东部产业新城、西部生态新区、绿色城南建设，着力完善配套、提升品味。加快建立城市管理标准化体系，推进联勤综合执法，提升管理精细化水平。继续开展市容市貌、超载超限、渣土运输等专项整治。加大土地经营力度，强化土地市场调控。
　　促进县城和集镇建设扩容提质。加快金寨新县城、霍山县城、叶集新城区建设步伐，支持舒城、霍邱县城规划建设中等城市。深入开展县城规划建设管理提升行动。积极引导社会资本参与县城供水供气及污水、垃圾处理设施建设运营。大力推进小城镇发展，加强道路管网、电力通讯等基础设施建设，完善教育、文化、医疗等公共服务设施，不断提升小城镇功能，努力打造一批特色鲜明的工业强镇、农业大镇、商贸重镇和旅游名镇。
　　推进美丽乡村建设。科学编制美丽乡村建设规划，进一步优化中心村布点规划。加快开展乡镇政府驻地整治建设，突出抓好治脏、治乱，加强基础设施建设和公共服务配套。持续推进中心村建设及自然村环境整治，健全长效管护机制，确保长治久美。加快发展特色产业，推动一二三产融合发展，培育壮大村级集体经济。加强农村精神文明建设，大力培育乡村文明新风，积极弘扬优秀传统文化。
　　（七）坚持精准扶贫，坚决打好脱贫攻坚战。
　　实施扶贫重点工程。实施产业扶贫工程，大力发展特色农业、乡村旅游、光伏产业、电子商务，鼓励支持自主创业、返乡创业，促进贫困人口稳定增收。实施基础设施建设扶贫工程，加快农村交通、水利、电网以及信息基础设施建设，推进易地扶贫搬迁和贫困户危房改造，切实改善生产生活条件。实施就业扶贫和智力扶贫工程，加强基层就业和社会保障服务设施建设，强化职业技能培训。实施健康扶贫和社保兜底扶贫工程，建立完善贫困人口医疗保险和救助、农村最低生活保障制度，实施扶贫线与低保线“两线合一”，逐步提高五保户、低保户保障标准。
　　创新扶贫开发机制。创新金融扶贫机制，充分发挥政策性金融导向作用，深化金融扶贫示范区建设试点。加大政府扶贫资金投入，改革扶贫资金用管机制，统筹利用扶贫、农业、水利、交通等专项资金，积极利用政府和社会资本合作（PPP）、政府购买服务等新型投资方式。探索资产收益扶贫模式，增加贫困人群财产性收入。完善定点帮扶和驻村扶贫工作队制度，定期选派优秀中青年干部挂职扶贫、驻村帮扶。大力倡导民营企业扶贫，支持各类社会组织从事扶贫开发，广泛动员个人参与扶贫，支持村民互助社发展。
　　压实脱贫工作责任。认真落实脱贫攻坚“市负责、县主体”的领导体制和片区为重点、精准到村到户到人的工作机制，实行党政一把手负总责，进一步细化和压实各级责任，层层签订脱贫攻坚责任书，真正形成上下贯通、横向到边、纵向到底的责任体系。加强贫困村基层组织建设，完善村级组织运转经费保障机制。健全脱贫攻坚工作考核机制，强化考核监督。
　　（八）保障和改善民生，促进社会和谐。
　　巩固提升民生工程。在实施好省定33项民生工程项目上，新增中心城区公共自行车、公共停车场建设等3个市级项目。加大财政资金投入力度，鼓励和吸引企业、社会组织、个人投入民生建设。完善民生投入激励约束和绩效评估机制，强化项目资金跟踪问效。加大住房保障工作力度，改造棚户区住房2.7万户；加强公租房管理，推进公租房保障货币化。继续做好7类“老字号”群体、上岸定居渔民生活保障工作。
　　做好就业和社会保障工作。完善就业服务，强化创业扶持，深入实施就业促进工程、大学生创业引领计划、农民工职业技能提升计划，帮扶失业和就业困难群体就业。认真落实各项社会保障和社会救助政策，突出抓好困难群众基本生活保障。提高企业退休职工基础养老金、优抚对象抚恤和生活补助、城乡低保对象补助水平。建立低保标准量化调整机制。建立健全城乡居民临时救助和重特大疾病医疗救助制度。加快推进社会救助“一门受理、协同办理”机制建设。大力推进社会养老服务体系建设。进一步提高机关事业单位基本工资标准，并建立正常调整和增长机制。
　　统筹发展各项社会事业。健全完善城乡公共文化基础设施，确保市图书档案馆、科技文化馆建成使用，开工建设市体育中心、市游泳馆，继续推进乡镇综合文化服务中心和农民文化乐园建设。支持皖西庐剧传承创新，完成全国第一次可移动文物普查。开展“中国书法城”创建活动，促进传统媒体和新兴媒体融合发展。继续实施学前教育第二个三年行动计划、全面改善农村义务教育薄弱学校五年规划，完成中心城区基础教育公办学校第一个三年行动计划，启动第二个三年行动计划，推进新一轮普通高中布局调整。加快职业教育发展，充分发挥大别山职教集团的引领、统筹、示范作用，推动中高职有效衔接。积极创造条件，加快义务教育均衡县（区）国家验收步伐。加强重大传染病防控，深入开展爱国卫生运动。组织实施全面两孩政策，提高生育服务管理水平，加强出生人口监测预测。广泛开展全民健身运动。切实做好第三次全国农业普查工作。扎实推进数字方志网馆库建设。实施扶残助残工程，促进残疾人家庭增收。认真做好统计、物价、气象、人防、地震、对台、档案、邮政、外事侨务、老龄、妇女儿童等工作。适应军改新形势，加强国防动员、民兵预备役工作，深入开展双拥共建，推进军民融合深度发展。
　　提升社会治理水平。加大普法宣传力度，全面启动“七五”普法。深入开展矛盾纠纷排查调处工作，推进人民调解、行政调解、司法调解衔接联动。加强流动人口和特殊人群服务管理，切实做好社区矫正和安置帮教工作。全面落实党的民族宗教政策，依法加强民族宗教事务管理。深化“平安六安”建设，依法严密防范和惩治各类违法犯罪活动。强化监管，防范化解金融风险。健全防灾减灾救灾体制，深入开展“全国综合减灾示范社区”创建活动。严格食品药品安全监管，保障人民群众身体健康和生命安全。全面落实安全生产责任制，深入开展 “铸安”行动，坚决遏制较大以上安全事故发生。
　　各位代表！
　　建设职能科学、权责法定、执法严明、公开公正、廉洁高效、守法诚信的法治政府，是各级政府肩负的重要职责。我们要进一步增强法治意识，强化法治思维，提高依法行政能力，在法治轨道上推动政府各项工作迈上新台阶。提升科学履职能力。全面履行政府职能，正确处理政府与市场、与社会的关系，进一步厘清政府权力的范围和边界，真正实现行政权力进清单、清单之外无权力，切实做到法定职责必须为、法无授权不可为。牢固树立法治意识，坚持领导干部带头学法、模范守法，善于运用法律手段管理经济和社会事务。落实政府决策机制，严格执行政府决策程序。强化责任意识，全面实施“三查三单”制度，确保政令畅通、令行禁止。加快建设高素质公务员队伍，深化政府效能建设。提升依法行政能力。严格依照法定权限和程序行使权力，自觉接受人大及其常委会依法监督，坚持重大决策出台前向人大报告，认真办理人大代表的议案和建议。主动接受政协民主监督，认真办理政协委员提案，广泛听取各民主党派、工商联和无党派人士意见。主动接受司法监督、舆论监督、公众监督。严格实行执法人员持证上岗和资格管理制度，大力推进法制监督平台建设。建立健全行政裁量权基准制度，规范裁量范围、种类、幅度。全面落实行政执法责任制，加强执法监督。完善行政复议制度，提升行政复议能力，强化复议监督。提升为民服务能力。拓展党的群众路线教育实践活动成果，自觉践行“三严三实”，坚持不懈纠“四风”、转作风。建立健全市、县（区）、乡（镇）三级政务服务机构和场所，逐步实现各类政务服务平台的连接和融合，形成分工清晰、各级联动、运行规范、管理一体、体验一致的政务服务网络。积极推进行政审批事项网上办理，实现实体大厅与网上审批并行，部分项目全流程网上审批。全面推进政务公开，创新公开方式，以公开促落实、促规范、促服务。提升拒腐防变能力。严格落实党风廉政建设责任制，认真履行“一岗双责”，全面加强惩治和预防腐败体系建设，坚决查处各类违纪违法案件。推进政府重大任务和重大项目全过程效能监察，加强政府采购资金、政府投资项目、国有土地使用权出让等管理与监督。强化公共资金、国有资产、国有资源和领导干部经济责任审计，实现审计监督全覆盖，完善结果运用机制，加大整改落实力度。严格落实中央八项规定、国务院“约法三章”，严控“三公”经费支出。
　　各位代表！
　　实现新发展任务艰巨，创造新业绩使命光荣。让我们紧密团结在以习近平同志为总书记的党中央周围，在中共六安市委的坚强领导下，增强忧患意识，强化责任担当，凝心聚力，开拓进取，真抓实干，久久为功，为建设幸福六安、全面建成小康社会而努力奋斗!
